# Geographical distribution of *Culicoides* (DIPTERA: CERATOPOGONIDAE) in mainland Portugal: Presence/absence modelling of vector and potential vector species

**DOI:** 10.1371/journal.pone.0180606

**Published:** 2017-07-06

**Authors:** David W. Ramilo, Telmo Nunes, Sara Madeira, Fernando Boinas, Isabel Pereira da Fonseca

**Affiliations:** Centre for Interdisciplinary Research in Animal Health (CIISA), Faculty of Veterinary Medicine, University of Lisbon, Lisbon, Portugal; Universita degli Studi di Camerino, ITALY

## Abstract

Vector-borne diseases are not only accounted responsible for their burden on human health-care systems, but also known to cause economic constraints to livestock and animal production. Animals are affected directly by the transmitted pathogens and indirectly when animal movement is restricted. Distribution of such diseases depends on climatic and social factors, namely, environmental changes, globalization, trade and unplanned urbanization. *Culicoides* biting midges are responsible for the transmission of several pathogenic agents with relevant economic impact. Due to a fragmentary knowledge of their ecology, occurrence is difficult to predict consequently, limiting the control of these arthropod vectors. In order to understand the distribution of *Culicoides* species, in mainland Portugal, data collected during the National Entomologic Surveillance Program for Bluetongue disease (2005–2013), were used for statistical evaluation. Logistic regression analysis was preformed and prediction maps (per season) were obtained for vector and potentially vector species. The variables used at the present study were selected from WorldClim (two climatic variables) and CORINE databases (twenty-two land cover variables). This work points to an opposite distribution of *C*. *imicola* and species from the Obsoletus group within mainland Portugal. Such findings are evidenced in autumn, with the former appearing in Central and Southern regions. Although appearing northwards, on summer and autumn, *C*. *newsteadi* reveals a similar distribution to *C*. *imicola*. The species *C*. *punctatus* appears in all Portuguese territory throughout the year. Contrary, *C*. *pulicaris* is poorly caught in all areas of mainland Portugal, being paradoxical present near coastal areas and higher altitude regions.

## Introduction

Vector-borne diseases (VBD) are influenced by complex ecological processes that regulate the distribution and abundance of vectors [1]. Historically, successful VBD prevention has relied upon management or elimination of vector populations from the environment [2]. In order to understand VBD dynamics it is crucial to recognize the influencing factors of all its components, particularly, the interactions occurring between vectors and their physical or biological environments. New technologies with the potential to improve our knowledge towards such relationships include remote sensing, geographic information systems and spatial statistics [3]. A broad range of data regarding vegetation, water, atmosphere, weather and land use is continuously collected by satellites, globally. Such information is available for research and may have its direct applications in VBD [4]. For instances, different environmental aspects associated with VBD [5] and different techniques, such as environmental niche modelling (ENM), can be used to understand the potential distribution of species involved in disease transmission, as well as to predict areas of potential risk [5–8].

*Culicoides* biting midges occur throughout most inhabited world, where they transmit a wide variety of pathogens of human and veterinary importance. Orbiviruses, such as Bluetongue virus (BTV), African Horse Sickness virus (AHSV) and Epizootic Haemorrhagic Disease virus (EHDV) are among the ones with most impact on animals [9,10]. Bluetongue disease (BTD) outbreaks alone are estimated to cause annual losses of approximately $3 billion, due to the morbidity and mortality of affected animals, trade embargoes and vaccination costs [11,12]. However, because BTV is transmitted by different *Culicoides* species and since there is a fragmentary knowledge of their ecology it is difficult to predict regional occurrence [13–15] making disease elimination in enzootic areas unfeasible [16].

In Europe, BTV has already been isolated from *C*. *imicola*, as well as from species from Obsoletus group (*C*. *obsoletus*, *C*. *scoticus*, *C*. *chiopterus*, *C*. *dewulfi* and *C*. *montanus* in Western Europe) and from *C*. *pulicaris* [17,18]. The genome of this virus has also been detected in parous females (with a complete reproductive cycle) of *C*. *newsteadi*, *C*. *punctatus*, *C*. *lupicaris* and species belonging to Nubeculosus complex [19–21].

After the 2004 Bluetongue outbreak that occurred in mainland Portugal, a National Entomologic Surveillance Program (NESP) for BTD was created. The capture of *Culicoides* biting midges from cattle, sheep, goats and horses farms was essential to characterize the distribution of *Culicoides* species in the country [22,23].

The present study sought to investigate distribution and probability models for the occurrence of vector and potential vector *Culicoides* species in mainland Portugal, per season, using data collected during NESP (2005–2013), using ENM. Results enlighten the presence/absence of vector and potential vector *Culicoides* species in mainland Portugal based on two climatic variables (WorldClim database) and twenty-two land cover variables (CORINE database), improving previously obtained data [23], while abundance studies are still ongoing. Variables with higher Pearson correlation coefficient (|r| >0.7) and with low impact on *Culicoides* occurrence near capture points were excluded. Findings are relevant for entomological and epidemiological surveillance actions, both necessary for risk assessment analysis and, ultimately, for the control of *Culicoides*-transmitted pathogens.

## Material and methods

### Ethics statement

Farm selection was performed by Direção Geral de Alimentação e Veterinária (DGAV), the Portuguese National Authority for Animal Health. All selected farms were privately owned and permissions were granted by the land-owners. Trap placement did not interfere with the livestock or with farm management. The material used in this study (midges from *Culicoides* genus) did not involved endangered or protected species.

### National Entomological Surveillance Program for BTD, insect sampling and morphological identification of *Culicoides* species

**Fig 1:**
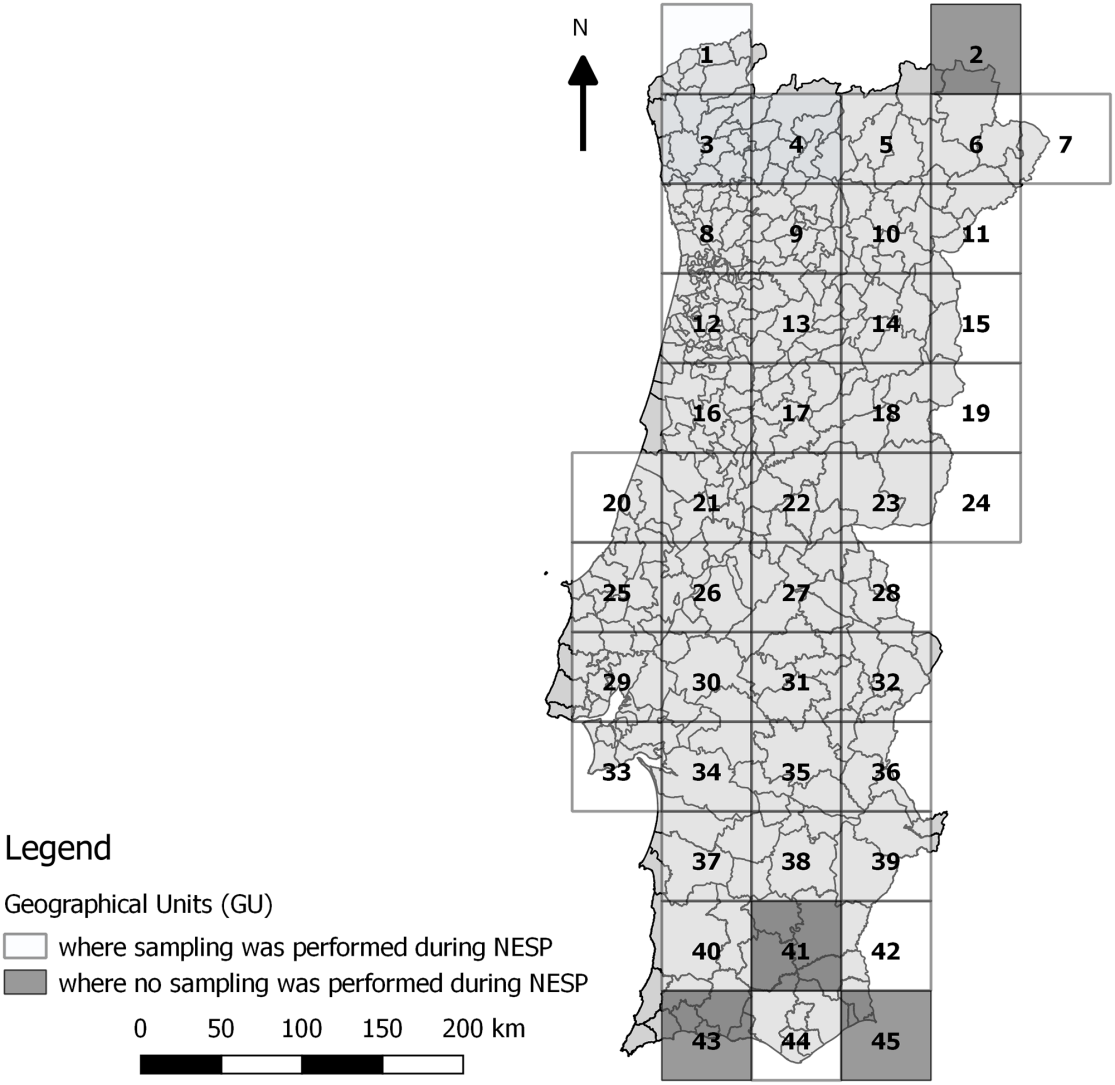
Geographical units in mainland Portugal.

To ensure systematic coverage during NESP, mainland Portugal was divided into 45 squares, named geographical units (GUs), each measuring 50 x 50 km (Fig 1).

Due to low livestock densities, four GUs were not sampled (2, 41, 43 and 45) because they were not considered to be of epidemiological interest (Fig 1). The selection of farms was performed as cited in [22].

Location of farms was obtained with Global Positioning System (GPS), allowing to localize farms geographically within the Nomenclature of Territorial Units for Statistics, subdivision 3 (NUTS III), where mainland Portugal is divided in 23 sub-regions (S1 Fig).

*Culicoides* were collected with CDC light traps (CDC miniature black light model 1212, John Hock, USA) fitted with 4 W UV bulbs, suction fans and LCS-2 Photoswitch systems. Traps were placed near animal enclosures (30 m) and 1.70 m above ground and operated from dusk to dawn, one night per week, throughout the year. Specimens were collected in flasks containing 75% of 70° ethanol and 25% of ethylene glycol as antifreeze, to a final volume of 500 ml.

*Culicoides* identification was performed using stereoscope microscopy (Olympus SZ51) and identified to species level by their wing pattern.

### Seasonal analysis

Collection calendar was defined in order to comprise the following schedule: spring (1^st^ March to 31^st^ May), summer (1^st^ June to 31^st^ August), autumn (1^st^ September to 30^th^ November) and winter (1^st^ December to 28^th^/29^th^ February). A separated analysis was performed for each season.

Due to the low number of captures performed throughout winter season, only 80 farms were selected, since 2005 to 2013. Still, for each of the other seasons (spring, summer and autumn) a total of 120 farms were selected for this study and the presence/absence of different *Culicoides* species during the 2005–2013 period was registered, for each farm and at each season.

### Environmental niche modelling

For *C*. *imicola*, Obsoletus group, *C*. *pulicaris*, *C*. *newsteadi* and *C*. *punctatus*, climatic and land cover variables were obtained from Bioclim (WorldClim—Global Climate Data, n.d.) (19 variables) and CORINE Land Cover (European Environment Agency, 1995) (44 variables) databases, respectively. CORINE Land Cover layers were processed using QGIS 2.10.1 software to produce maps with the minimum distance to each of the land cover classes. These maps and the information gathered by the presence (captured specimens) and absence (no captured specimens) of the referred *Culicoides* species at different seasons in mainland Portugal (S1–S5 Tables), were subjected to statistical analyses using R Studio^®^ software. The following steps were performed:

1. The correlation between the 63 variables was evaluated and the variables with higher Pearson correlation coefficient (|r| >0.7) and low impact on *Culicoides* occurrence near capture points were excluded to avoid collinearity in the final model. Table 1 shows the variables chosen for this work. Due to the high correlation between climate variables, only two of them (mean temperature of the wettest quarter of the year and mean temperature of the driest quarter of the year) were selected to be included in the model. Twenty-two land cover variables were also selected, excluding those with low relation with rural and farm areas, where traps were placed.
2. For each species at each season, an univariate logistic analysis was performed taking into account 75% of the records randomly chosen (90 collecting points for spring, summer and fall and 60 for winter), and those that were statistically significant (*p*-value below a pre-defined threshold of *p* <0.1) with the response variable (probability for that species to occur in that season and region) were selected.
3. Variables that did not improve the final model, i.e. with a higher Akaike Information Criterion (AIC) value, were excluded, using a backward-forward variable selection procedure, and a multivariate logistic regression model was obtained.
4. Finally, cut-off, sensitivity and specificity values were defined from the Receiving Operating Curve (ROC) elaborated with the remaining 25% of the collecting points that were not included in the first analysis. The cut-off point was defined to consider when a *Culicoides* species is effectively present or absent, with a respective level of sensitivity and specificity.
5. Probability maps concerning species presence or absence were elaborated after model validation mentioned above, based in the following mathematical expression: $\text{Y}=\text{log}\left(\frac{P}{A}\right)=\;\alpha +\;\beta i\chi i$ where *Y* is the response variable (probability of a species to be present in a determined area), *P* is the species presence, *A* is the species absence, *α* is a coefficient representing the intersection value with *Y* axis when *X* is zero, *χi* is the variable (climate or land cover type) value in one specific point and *βi* the coefficient of that respective variable.

**Table 1:** Climate and land cover variables chosen for model analysis.

Variable type	Variable code	Description
Climate (n = 2)	Bio8	Mean temperature of wettest quarter of the year
	Bio9	Mean temperature of driest quarter of the year
Land cover (n = 22)	111	Continuous urban fabric
	112	Discontinuous urban fabric
	132	Dump sites
	133	Construction sites
	141	Green urban areas
	211	Non-irrigated arable land
	212	Permanently irrigated land
	222	Fruit trees and berry plantations
	223	Olive groves
	231	Pastures
	244	Agro-forestry areas
	311	Broad-leaved forest
	312	Coniferous forest
	313	Mixed forest
	321	Natural grassland
	322	Moors and heathland
	411	Inland marshes
	412	Peatbogs
	421	Salt marshes
	511	Water courses
	512	Water bodies
	523	Sea and ocean

## Results

### Influence of selected variables

Variables that influenced positively or negatively the occurrence of referred *Culicoides* species per season are represented in Tables 2–6. For the land cover variable, a positive value corresponds to a lower probability for a species to occur when the variable is closer to the capture point, while for a climate variable, a positive value means that higher temperatures favour midges’ occurrence.

**Table 2:** Variables influencing (p<0.05) the occurrence of *C*. *imicola*, per season (2005–2013).

*Culicoides* species	Season	Variables	β value	α value
*C*. *imicola*	Spring	Mean temperature of wettest quarter	0.0301	-9.2993
		Mean temperature of driest quarter	0.0320	
		Non-irrigated arable land	-0.0002	
	Summer	Mean temperature of driest quarter	0.1032	-21.72
		Permanently irrigated land	-9.561x10^-5^	
	Autumn	Mean temperature of wettest quarter	0.0966	-45.90
		Mean temperature of driest quarter	0.1726	
		Construction sites	1.141x10^-4^	
		Non-irrigated arable land	-2.487x10^-4^	
		Fruit trees and berry plantations	-7.809x10^-5^	
		Olive groves	4.719x10^-5^	
		Moors and heathland	-5.067x10^-5^	
	Winter	Mean temperature of driest quarter	0.0601	-13.86
		Discontinuous urban fabric	2.595x10^-4^	
		Water bodies	-1.416x10^-4^	

α value: Y value when X is zero; β value: variable coefficient.

**Table 3:** Variables influencing (p<0.05) the occurrence of Obsoletus group species, per season (2005–2013).

*Culicoides* species	Season	Variables	β value	α value
Obsoletus group	Spring	Non-irrigated arable land	3.951x10^-4^	0.6866
		Agro-forestry areas	1.062x10^-4^	
	Summer	Agro-forestry areas	3.356x10^-5^	1.136
		Natural grassland	-4.760x10^-5^	
	Autumn	Mean temperature of wettest quarter	-0.0192	15.4369
		Mean temperature of driest quarter	-0.0605	
	Winter	Pastures	4.861x10^-5^	-1.080
		Agro-forestry areas	2.875x10^-5^	
		Water bodies	7.156x10^-5^	

α value: Y value when X is zero; β value: variable coefficient.

**Table 4:** Variables influencing (p<0.05) the occurrence of *C*. *pulicaris* per season (2005–2013).

*Culicoides* species	Season	Variables	β value	α value
*C*. *pulicaris*	Spring	Mean temperature of driest quarter	-0.0276	6.940
		Dump sites	-1.438x10^-5^	
		Mixed forest	-1.055x10^-4^	
		Natural grassland	-8.173x10^-5^	
	Summer	Pastures	-7.218x10^-5^	0.3997
		Agro-forestry areas	2.581x10^-5^	
		Mixed forest	-1.387x10^-4^	
	Autumn	Fruit trees and berry plantations	4.069x10^-5^	-1.497
		Broad-leaved forest	2.314x10^-4^	
		Mixed forest	-1.328x10^-4^	
	Winter	Mean temperature of driest quarter	-0.0599	10.7438
		Broad-leaved forest	0.0002	

α value: Y value when X is zero; β value: variable coefficient.

**Table 5:** Variables influencing (p<0.05) *C*. *punctatus* occurrence, per season (2005–2013).

*Culicoides* species	Season	Variables	β value	α value
*C*. *punctatus*	Spring	Natural grassland	-6.126x10^-5^	2.638
	Summer	Inland marshes	1.058x10^-5^	0.6107
	Autumn	Construction sites	2.310x10^-5^	0.3572
	Winter	Construction sites	3.5x10^-5^	0.2163

α value: Y value when X is zero; β value: variable coefficient.

**Table 6:** Variables influencing (p<0.05) *C*. *newsteadi* occurrence, per season (2005–2013).

*Culicoides* species	Season	Variables	β value	α value
*C*. *newsteadi*	Spring	Mean temperature of wettest quarter	0.0367	-2.225
		Agro-forestry areas	-3.302x10^-5^	
	Summer	Agro-forestry areas	-3.961x10^-5^	1.141
		Natural grassland	1.097x10^-4^	
		Water courses	-3.509x10^-5^	
	Autumn	Mean temperature of wettest quarter	0.042	-0.7937
		Discontinuous urban fabric	-2.443x10^-4^	
		Olive grves	-4.442x10^-5^	
		Agro-forestry areas	-3.898x10^-5^	
		Water courses	-2.597x10^-5^	
	Winter	Mean temperature of wettest quarter	0.0459	-4.553
		Olive groves	-4.256x10^5^	
		Coniferous forest	1.413x10^-4^	

α value: Y value when X is zero; β value: coefficient of the variable.

A broad perspective of the variables that influence the occurrence of different *Culicoides* species is present on Table 7.

**Table 7:** Summary of variables that influence (p<0.05) the occurrence of different *Culicoides* species per season.

	*C*. *imicola*				Obsoletus group				*C*. *pulicaris*				*C*. *punctatus*				*C*. *newsteadi*			
	Sp	Su	Au	Wi	Sp	Su	Au	Wi	Sp	Su	Au	Wi	Sp	Su	Au	Wi	Sp	Su	Au	Wi
Bio8																				
Bio9																				
111																				
112																				
132																				
133																				
141																				
211																				
212																				
222																				
223																				
231																				
244																				
311																				
312																				
313																				
321																				
322																				
411																				
412																				
421																				
511																				
512																				
523																				

Sp: Spring; Su: Summer; Au: Autumn; Wi: Winter. Red colour: negative correlation; Green colour: positive correlation. Blank space: no significant variable.

With the exception of *C*. *punctatus*, all analysed *Culicoides* species occurrence demonstrated to be influenced by climatic variables at, at least, in one season. *C*. *imicola* and *C*. *newsteadi* species presence are dependent of high mean temperatures. *C*. *imicola* is mostly influenced by high mean temperatures during the driest quarter of the year, at all seasons; while *C*. *newsteadi* is influenced by high mean temperatures in the wettest quarter of the year (spring, autumn and winter). *C*. *pulicaris* and species from the Obsoletus group are influenced by low mean temperatures, especially, in the wettest quarter of the year (autumn, spring and winter).

For all analysed species, several land cover variables have influenced positively or negatively their occurrence at different seasons. *C*. *imicola* species presence is mostly positively influenced by agricultural areas (non-irrigated and permanently irrigated land, fruit trees and berry plantations). Obsoletus group species and *C*. *pulicaris* are negatively influenced by agro-forestry areas (annual crops or grazing land under the wooded cover of forestry species), while *C*. *newsteadi* presence is dependent on agro-forestry areas, as well as water courses (natural or artificial, serving as water drainage channels, with a minimum width of 100 m). The presence of mixed forest (trees, including shrub and bush understories, where broad-leaved and coniferous species co-dominate) is important for *C*. *pulicaris* to occur at different seasons. Broad-leaved forest does not seem to beneficiate this species. Construction sites (including soil or bedrock excavations and earthworks) do not favour *C*. *punctatus* occurrence at autumn nor at winter seasons.

### Cut-off points and presence/absence probability maps

The cut-off points represented in Table 8 were obtained based on the highest combination of sensitivity and specificity values according to the presence/absence of *Culicoides* species in a specific area per each season.

**Table 8:** Cut-off points with the correspondent sensitivity and specificity mean values for each species per each season.

*Culicoides* species	Season	Cut-off point	Sensitivity (%)	Specificity (%)
*C*. *imicola*	Spring	0.6592	80	82.86
	Summer	0.6254	89.29	81.25
	Autumn	0.6864	95	100
	Winter	0.5959	57.14	100
Obsoletus group	Spring	0.6835	70.83	83.33
	Summer	0.6782	68	66.67
	Autumn	0.6475	84.21	90
	Winter	0.6407	76.92	57.14
*C*. *pulicaris*	Spring	0.6018	55.56	66.67
	Summer	0.5679	77.78	57.14
	Autumn	0.6131	40	90
	Winter	0.5465	100	87.5
*C*. *punctatus*	Spring	0.7160	82.61	42.86
	Summer	0.6952	66.67	85.71
	Autumn	0.6646	65	77.78
	Winter	0.6927	56.25	75
*C*. *newsteadi*	Spring	0.6526	78.26	85.71
	Summer	0.6785	50	100
	Autumn	0.66	83.33	66.67
	Winter	0.66	76.92	100

**Fig 2:**
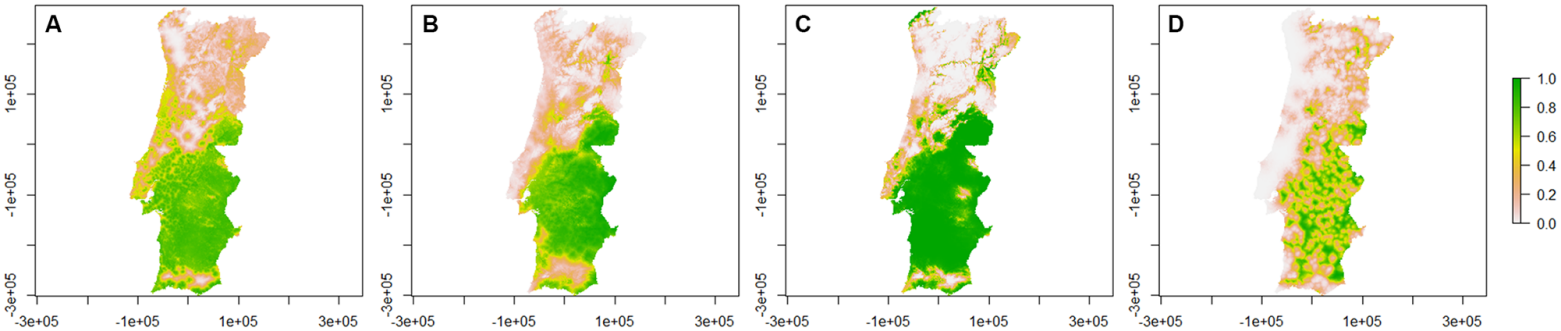
Presence/absence probability maps for *C*. *imicola* per season. A—Spring; B—Summer; C—Autumn; D—Winter. Bar: probability of presence/absence of *C*. *imicola*.

The presence/absence probability maps for *C*. *imicola* obtained from the models are represented in Fig 2. and regions names are in S1 Fig.

There is a high probability for *C*. *imicola* to appear below Centro region and in Beira Baixa from spring to autumn, but low in winter. In the remaining territory, as well as in the areas of Alentejo Litoral, Baixo Alentejo and Algarve, the probability for *C*. *imicola* presence is very low. *C*. *imicola* has a higher probability of occurrence at autumn.

**Fig 3:**
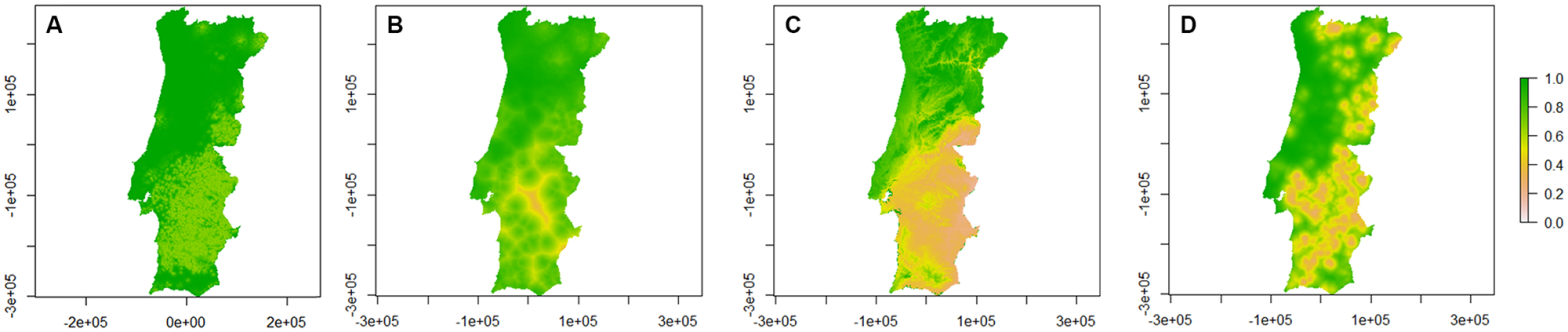
Presence/absence probability maps for Obsoletus group species per season. A—Spring; B—Summer; C—Autumn; D—Winter. Bar: probability of presence/absence of Obsoletus group species.

The presence/absence probability maps for Obsoletus group species obtained from the models are represented in Fig 3.

Midges from Obsoletus group demonstrate a similar distribution in all territory at spring and summer, being, however, less probable to be found in Alentejo region. In autumn, the probability to collect specimens of Obsoletus group species falls abruptly below Centro region, contrary to *C*. *imicola*. In winter, Obsoletus group species are less probable to be found in Alentejo region and in eastern areas of Norte and Centro. *Culicoides* midges from Obsoletus group are more common during spring and summer in areas above Alentejo region.

**Fig 4:**
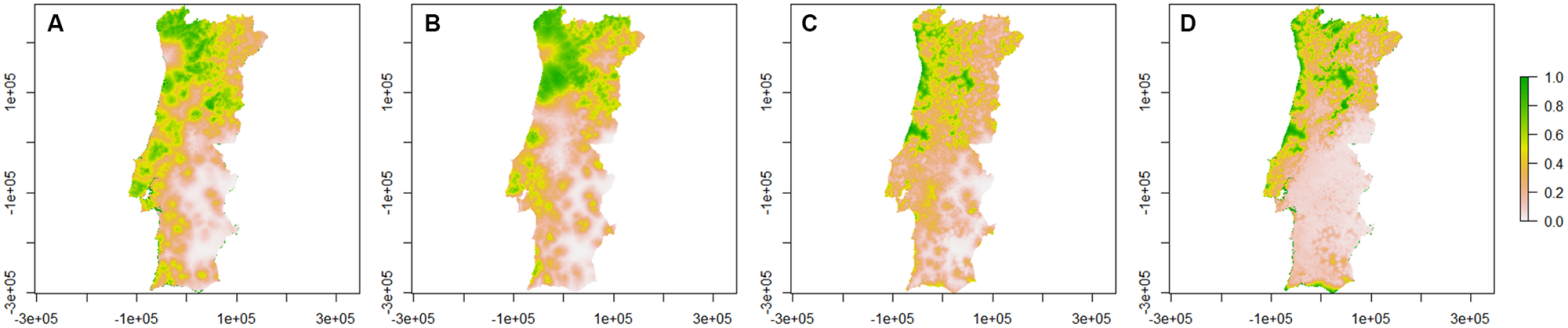
Presence/absence probability maps for *C*. *pulicaris* per season. A—Spring; B—Summer; C—Autumn; D—Winter. Bar: probability of presence/absence of *C*. *pulicaris*.

The presence/absence probability maps for *C*. *pulicaris* obtained from the models are represented in Fig 4.

*C*. *pulicaris* appears in both coastal and inland regions of mainland Portugal from the North to Área Metropolitana de Lisboa. In Alentejo, the probability to collect this species is extremely low. In spring, it has a high probability to appear from Alto Minho to Região de Aveiro. In winter, this species concentrates in regions near Atlantic Ocean, with the exception of the Alentejo coast. In all seasons, *C*. *pulicaris* tend to appear in high altitude regions from Viseu-Dão-Lafões and near Beiras and Serra da Estrela.

**Fig 5:**
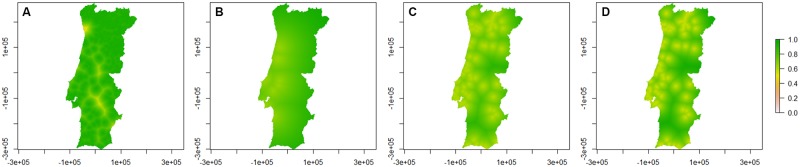
Presence/absence probability maps for *C*. *punctatus* per season. A—Spring; B—Summer; C—Autumn; D—Winter. Bar: probability of presence/absence of *C*. *punctatus*.

The presence/absence probability maps for *C*. *punctatus* obtained from the models are represented in Fig 5.

*C*. *punctatus* almost has an equal distribution, in mainland Portugal, throughout the year, being less probable to find in Área Metropolitana do Porto during spring. Still, autumn and winter probability maps are very similar.

**Fig 6:**
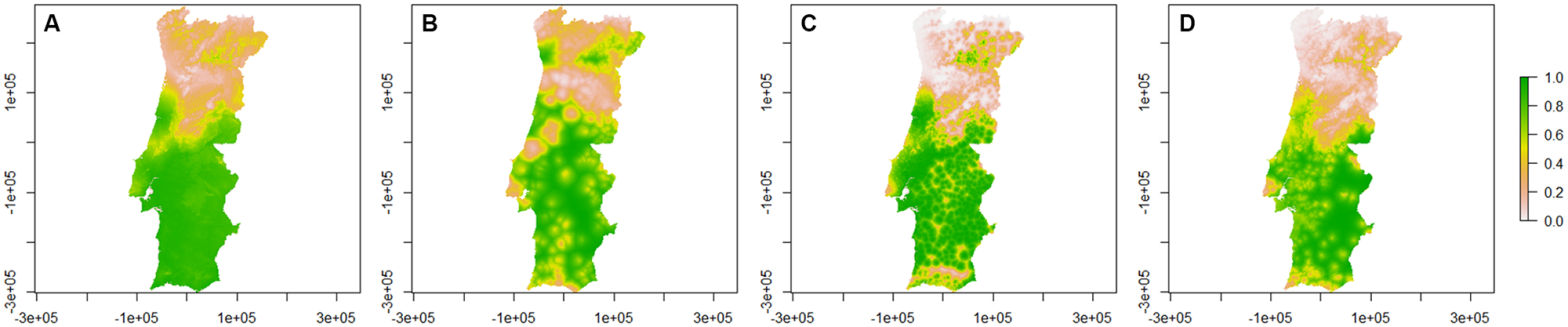
Presence/absence probability maps for *C*. *newsteadi* per season. A—Spring; B—Summer; C—Autumn; D—Winter. Bar: probability of presence/absence of *C*. *newsteadi*.

The presence/absence probability maps for *C*. *newsteadi* obtained from the models are represented in Fig 6.

*C*. *newsteadi* has a similar distribution in spring, autumn and winter, being common in Alentejo and Algarve, as well as in Beira Baixa and at the coastal area of Região de Aveiro to Região de Leiria. This species is not common at Norte region throughout these three seasons but during the summer the pattern is slightly different, with a small concentration near the coastal areas of Área Metropolitana do Porto and Douro. Although *C*. *newsteadi* is present all year, it is more common to appear during spring.

## Discussion

### *C*. *imicola*

High mean temperatures are important for the occurrence of *C*. *imicola* in the studied territory throughout the year. This species can also be found in other geographical areas where high temperatures and dry environments occur, like African, Middle Eastern and Southeast Asian countries [24].

During spring, the existence of non-irrigated arable land favours this species appearance, probably due to the development of their pupal phases which would not survive in aquatic environments [25,26], restricting this species to flat and slow-draining regions with clay soils (nutrient-rich, water-holding soil) [27,28].

This species was not captured with relative humidity below 9% and temperatures above 40°C (S6 Table). The presence of permanently arable land with water sources nearby, is favourable for this species development in the summer. However, high temperatures (≥40°C) combined with elevated dryness (typical from certain areas of Alentejo region) are fatal [28–30]. Rapid soil surface layer desiccation has also a negative impact in *C*. *imicola* occurrence [28,31], since its absence has been associated with soils with a sandy texture, known to have depleted moisture levels (especially in the surface layer) and therefore lacking vital nutrients for *C*. *imicola* survival [28].

In mainland Portugal, during autumn, non-irrigated arable land, permanent crops, moors and heathland areas (bushes, shrubs and herbaceous plants) are suitable for *C*. *imicola*. This shows its preference for different trees and, again, for drier environments used, probably, for breeding. This species breeds in areas where sunny surfaces prevail together with low vegetation [27]. *C*. *imicola* avoid areas covered by forest diverging from other European vector species, as those belonging to Obsoletus complex [28,32]. The negative influence of some permanent crops (olive groves) in *C*. *imicola* occurrence shows that this species may have preferences when choosing the best vegetation for breeding, oviposition and for larval and pupae development, which must be further evaluated. Terrains with human involvement (construction sites) diminish the risk for this species occurrence.

*Culicoides* vector activities usually reduce or even cease at low temperatures and BTV transmission, in many temperate regions, is interrupted for several months by cold weather. Nevertheless, virus overwintering may result in outbreaks [24]. The appearance of *C*. *imicola* near inland waters during winter must be further investigated, since these environments could represent a preferential place for virus overwintering.

*C*. *imicola* species are concentrated in regions below Centro region and in Beira Baixa. The low probability of occurrence observed in a small region between Alentejo Litoral, Baixo Alentejo and Algarve is probably due to the very dry climate and hot temperatures registered in this region, especially in summer. The geographical range of *C*. *imicola* appears primarily limited by cold and dry stresses, and to a lesser extent, by wet stress [29]. In Spain, *C*. *imicola* has established itself in all southwest (near the Portuguese frontier) and central regions of Madrid province. In the Mediterranean zone it has been found on the coast of Catalonia, as well as near Alicante and Murcia [33]. It is also a largely abundant species in the Balearic Islands [34]. The large-scale distribution pattern appears to be strongly influenced by species-specific requirements for high temperatures and dry summers [35].

It is important to refer that, during autumn, *C*. *imicola* has the capacity to disperse further North than at other seasons. Although the estimated cut-off point for this season was high (0.69, with a mean sensitivity of 95% and a mean specificity of 100%), the occurrence of this species in Alto Minho and Douro regions can be expected (Fig 2), as it was pointed out in previous works [36,37]. In other communities, especially in Castile and North zone, there have been sporadic incursions of specimens, which do not prevail [33], as well as previously observed by other authors within the Portuguese territory [23,36,37]. It is possible that *C*. *imicola* is expanding its range northwards, due to climate changes [35]. However, several factors, such as dispersal abilities, size of the source population, meteorological conditions and the presence of natural barriers, limit colonization [29].

### Obsoletus group species

Agro-forestry areas do not favour the occurrence of Obsoletus group species near capture points. These species probably tend to remain close to their preferred habitats (shaded areas in pastures, near woodlands) instead of searching for stabled animals, only feeding on them when they are at pastures and not at enclosed stables. Thus, more preferential hosts exist in wild fauna and they are probably substitutes for their blood meals, in agreement with previous works [38]. Talavera et al. [39] have also shown that the same main BTV vector species that were present on farms were also present on neighbouring natural areas along with wild ruminants. Such findings support their putative role as bridge vectors for arboviruses transmission between wild and domestic ruminants, in addition to their recognised role as epizootic vectors. The transmission among wild/domestic communities by Culicoides bridge vectors (*C*. *imicola* and Obsoletus group) could facilitate BTV reintroduction among domestic ruminants.

Natural grassland close to capture points act as a contributing variable for these species occurrence, probably due to the presence of animal manure, which provide an optimal environment for larvae development in the summer. Although some studies concerning overwintering of Obsoletus group species [8,28] were performed, further research is needed to understand the importance of agro-forestry, pastures and water bodies in the absence of Obsoletus group species near capture points in winter.

In what concerns climatic variables, low mean temperatures in both wet and dry quarters of the year raises the probability of these species occurrence in autumn, in agreement with their preferences regarding their spatial distribution (mainly in Central and Northern European countries) [24,28]. Midges from Obsoletus group demonstrate a high tolerance for a wide range of temperatures, altitudes and terrain slopes, having a broad distribution in the European continent [28]. Species from Obsoletus complex only occur within the temperate and boreal ecozones [40] of the Holarctic region (includes Palearctic and Nearctic ecozones), despite some species have ability to penetrate southwards into the northern half of the Mediterranean region [41].

Species from Obsoletus group prefer Norte and Centro mainland Portugal regions, being almost absent in Alentejo during autumn and winter. Although *C*. *imicola* and Obsoletus group species have some overlapping or common areas of occurrence, they show different preferences in autumn and winter seasons, having their occurrence in mainland Portugal almost opposite. This distribution has been explained by the fact that *C*. *imicola* is present in the warmest zones, while species belonging to Obsoletus group require areas with a relatively low annual average temperatures and high soil moisture [33,35,42].

### *C*. *pulicaris*

*C*. *pulicaris* seems to be less adaptable to the Portuguese environmental conditions when compared to the other studied species (S7 Table). *C*. *pulicaris* larvae were already collected from molehill soil; silt from the edge of a pond; maize silages reserves; soil in stagnant water; algae and underlying soil; river edges; forest mud; wet grazed field with manure; waterlogged soils near lakes and marshy places and forest leaf litters [14,43–49]. It can be pointed out that different kinds of vegetation influence their presence or absence, near the capture points. This species tend to remain in their preferred habitats (broad-leaved forest, agro-forestry areas, permanent crops), where they probably feed on wild fauna and not on farm animals or even may overwinter, similarly to Obsoletus group species. This could justify why *C*. *pulicaris* is less captured. Still, it must be referred that mixed forest, natural grassland and pastures nearby capture points also raises this species occurrence, which must be further investigated. Additionally, this species preference for human made structures, may partially justify its occurrence near urban areas, that may be used for larval and pupal development or to make a human blood meal, as reported by other authors [50]. This species occurrence is favoured by lower mean temperatures in the driest quarter of the year (spring and winter), as Obsoletus group species. This fact justifies the similar spatial distributions to Obsoletus group species, across Europe [51].

*C*. *pulicaris* is well distributed by several European countries, being its geographic range similar to species from Obsoletus group [33,51]. This species has a preference for Northern areas, being more probably found at coastal regions and also at higher altitude zones. However, Pena [36] referred its presence mainly in the Northeast and in the Southeast regions, near the Spanish border. Although with low abundance, *C*. *pulicaris* is also dispersed by all Spanish territory, being more commonly found at the Southern zone of Iberia than at the North [33]. Probably it is not limited to a specific ecosystem, being present in several types of environmental and ecological conditions that need be further investigated.

### *C*. *punctatus*

*C*. *punctatus* is the most well adapted species to Portuguese environment. It is present all year and, according to our results, only few variables influence its occurrence near the capture sites. *C*. *punctatus* larvae have been found at the same places as *C*. *pulicaris*, in open marshy fields [52], in sludge samples, with or without organic matter or animal manure and together with *C*. *imicola* [36]; in wet soil between silage reserves (together with *C*. *stigma*), soil in stagnant water, algae and underlying soil [14] and silt from a pond [13,53]. Most importantly, it can be observed that *C*. *punctatus* occurrence is not favoured by human presence. This study shows that wetlands do not seem to favour this species occurrence (at least in the summer), contrarily to what previous works show [14,52].

*C*. *punctatus* is well distributed in Europe, from Ireland to Russia [33] being found in Palearctic ecozone until Mongolia, Near East and North of Africa, as well as in the African Tropical zone, appearing well adapted to these geographical regions. This work shows that this species is dispersed around Portugal mainland, in agreement with Pena [36]. The species *C*. *punctatus* has a 50% or more probability of occurrence at any part of the Portuguese mainland, evidencing how well adapted it is to its climate.

### *C*. *newsteadi*

*C*. *newsteadi* is influenced by higher mean temperatures at the wettest quarter of the year, like *C*. *imicola* species. *C*. *newsteadi* shows an intermediate distribution between *C*. *punctatus* and *C*. *pulicaris*.

Like for most of *Culicoides* species, the breeding sites of *C*. *newsteadi* are also poorly known. Several types of vegetation favour this species occurrence, like agro-forestry areas, permanent crops, as well as water courses. However, forests and semi-natural areas (natural grasslands and coniferous forests) do not. Thus, this species shows vegetation preferences, as well as *C*. *imicola*. Human made structures favour this species occurrence in autumn, what reinforces what has been shown in previous works concerning *C*. *newsteadi* feeding preferences [54,55]. Additionally, this species has already been reported breeding in shallow, brackish pools, lined with decaying vegetable material [25,39]. Pena [36] also recovered this species in mud samples from mainland Portugal.

Our results show that *C*. *newsteadi* mimics *C*. *imicola* species distribution pattern, although it can be found at northern regions, in agreement with Pena [36]. Its absence in Norte and Centro regions is probably due to more adverse climatic conditions during the colder seasons, as it happens in most of central and eastern European from where it is also absent [33].

### Conclusion

Knowledge of suitable breeding sites of each species, particularly from those implicated in the transmission of parasites or pathogens, is essential to predict areas of potential risk and with it to contribute to the development of new integrated control strategies. Climatic variables may not be a limiting factor for species occurrence, since presence/absence of breeding sites may also play a role inside microclimatic conditions.

This work reveals that several climatic and land cover variables differently influence *Culicoides* presence/absence in mainland Portugal. However, the impact of each variable in *Culicoides* behaviour should be further analysed

Since there is a fragmentary knowledge concerning *Culicoides* ecology there is always a degree of uncertainty in the modelled results. Nevertheless, presented data constitute valuable auxiliary information to entomological and epidemiological surveillances as control measures to reduce the risk of outbreaks.

## Supporting information

S1 Fig Nomenclature of territorial units for statistics, subdivision 3. (TIF)

S1 Table Distribution of captured *C*. *imicola* specimens per season (2005–2013). (PDF)

S2 Table Distribution of captured Obsoletus group specimens per season (2005–2013). (PDF)

S3 Table Distribution of captured *C*. *pulicaris* specimens per season (2005–2013). (PDF)

S4 Table Distribution of captured *C*. *punctatus* specimens per season (2005–2013). (PDF)

S5 Table Distribution of captured *C*. *newsteadi* specimens per season (2005–2013). (PDF)

S6 Table Meteorological data obtained from the closest meteorological stations to the farms. (PDF)

S7 Table Absolute and relative frequencies of estimated and analysed *Culicoides* collected during the NESP for BTD (2005–2013) in mainland Portugal. (PDF)
