# Supplementary material for: Geographical distribution of Culicoides (DIPTERA: CERATOPOGONIDAE) in mainland Portugal: Presence/absence modelling of vector and potential vector species
Source: PLoS One. 2017 Jul 6;12(7):e0180606. doi: 10.1371/journal.pone.0180606 (PMC5500329; doi:10.1371/journal.pone.0180606)
Supplement: S3 Table — (PDF) [file pone.0180606.s004.pdf]

S3 Table – Distribution of captured *C. pulicaris* specimens per season (2005-2013)

Capture Points	Latitude	Longitude	Spring	Summer	Autumn	Winter	Code
AFM79	41,91685	-8,57085	1	1	1	1	Many
AG0E1	41,8237	-8,49156	1	1	1	N/A	10
AH00J	40,14665	-7,50982	1	N/A	N/A	N/A	10
ASZ44	41,53135	-8,61674	1	1	1	0	Many
AYR11	41,41411	-8,52316	0	N/A	N/A	N/A	1
AYR16	41,41411	-8,52316	0	N/A	N/A	N/A	1
BEL34	41,34332	-8,48753	0	N/A	N/A	N/A	10
BEN10	41,34332	-8,48753	0	N/A	N/A	N/A	10
BKC70	41,26538	-8,00113	1	1	0	0	Many
BKF33	41,27366	-8,08692	N/A	N/A	1	1	Many
BP6A3	41,13517	-8,1315	N/A	0	0	N/A	10
BVC08	41,02351	-8,32525	0	1	1	1	Many
CCG15	41,53135	-8,61674	0	N/A	N/A	N/A	10
EA01R	41,71617	-7,76775	1	1	1	N/A	Many
EA73M	41,8584	-7,78437	0	N/A	N/A	N/A	1
EA97H	41,8175	-7,77556	N/A	0	N/A	N/A	1
EAC39	41,70917	-8,05387	N/A	1	1	N/A	10
EB6J8	41,61198	-7,56206	N/A	1	N/A	N/A	1
EBQ94	41,78055	-7,48233	N/A	N/A	1	N/A	1
ED18F	41,63513	-6,8164	N/A	N/A	1	N/A	1
EE23H	41,70324	-7,7845	N/A	N/A	0	N/A	1
EE8E8	41,68215	-7,81274	N/A	0	N/A	N/A	1
EE91A	41,71047	-7,69161	N/A	0	N/A	N/A	1
EF15G	41,54339	-7,54594	N/A	1	N/A	N/A	1

EG50H	41,58482	-7,30148	N/A	0	N/A	N/A	1
EH72D	41,5092	-7,16503	1	1	0	1	Many
EH81G	41,47649	-7,17788	N/A	N/A	0	N/A	1
EL04D	41,5103	-6,63	N/A	0	N/A	N/A	1
EL42E	41,565	-6,622	1	0	1	1	Many
EL44F	41,51377	-6,40468	0	0	N/A	N/A	10
EM57I	41,39959	-6,43948	0	1	0	0	Many
EM61H	41,52195	-6,26765	0	N/A	N/A	N/A	1
EM83M	41,38204	-6,42639	0	1	1	1	Many
ER29B	41,31806	-7,48361	N/A	0	0	N/A	10
EY21B	41,09392	-6,82373	0	1	0	0	Many
FF04B	41,05797	-7,09455	1	1	1	0	Many
HEG41	40,94123	-7,9893	N/A	N/A	0	N/A	1
HR94V	40,68969	-8,57343	1	1	1	0	Many
HV55E	40,46209	-8,00954	0	0	0	0	Many
HV91N	40,78414	-8,13037	N/A	N/A	0	N/A	1
HW4O3	40,66711	-7,87343	N/A	N/A	0	0	10
JE93C	40,45654	-8,44208	1	0	1	0	Many
JFC55	40,52674	-8,03798	0	0	0	0	Many
JG71D	40,51016	-7,95845	0	0	0	0	Many
JK78H	40,39833	-8,06667	0	0	0	0	Many
JT0D5	40,1667	-8,6	1	0	0	N/A	Many
JU57A	40,2118	-8,43002	0	0	0	0	Many
KA62I	40,15967	-8,27055	N/A	N/A	0	N/A	1
KD09K	40,15322	-8,67363	0	0	1	1	10
KD42O	40,01667	-8,61667	1	N/A	N/A	0	10
MB23E	40,77523	-6,97049	0	0	0	N/A	Many

ME22L	40,56638	-6,99404	N/A	0	0	0	10
MN10V	40,39272	-7,20398	0	N/A	N/A	N/A	1
MN82J	40,38789	-7,25184	0	0	0	N/A	10
MN84J	40,38789	-7,25184	1	1	0	N/A	Many
MP18E	40,25389	-7,475	0	0	N/A	0	10
MP19E	40,28333	-7,46472	1	0	1	1	10
MP23E	40,26528	-7,465	0	0	N/A	0	10
MP33E	40,25477	-7,47923	1	1	0	0	Many
MP63N	40,19091	-7,64074	N/A	N/A	0	0	10
MR16V	40,0134	-7,46114	0	0	0	0	Many
MR19K	40,16914	-7,47359	0	1	0	0	Many
MR67K	40,15875	-7,45567	N/A	0	0	N/A	10
MRN33	40,14665	-7,50982	0	1	1	N/A	Many
MRW94	40,09694	-7,42222	1	N/A	0	1	10
MS96J	40,1721	-7,26231	N/A	N/A	0	N/A	1
MS98G	40,22931	-7,14747	1	N/A	1	N/A	10
MV09I	39,9232	-7,29945	0	0	N/A	0	Many
MV13A	39,89214	-6,97247	1	0	0	0	Many
MV15C	39,89129	-7,28707	0	0	0	1	Many
MV24E	39,85615	-7,27472	N/A	N/A	N/A	0	1
MV31I	39,96842	-7,2975	0	N/A	N/A	N/A	1
MV34D	39,8448	-7,50147	0	N/A	0	N/A	10
MV46J	40,17211	-7,26231	N/A	0	1	N/A	10
MV51C	39,90752	-7,21071	0	N/A	N/A	N/A	1
MV71E	39,8715	-7,30444	0	0	1	0	Many
MV83E	39,83388	-7,33623	0	N/A	N/A	N/A	1
MV9C3	39,89434	-7,27339	0	0	0	N/A	Many

MW54M	39,76342	-8,04388	N/A	N/A	N/A	0	10
MW57N	39,77987	-8,76262	0	0	N/A	N/A	10
MY01A	39,66978	-7,64719	0	1	1	0	Many
MY17C	39,69924	-7,58914	0	N/A	N/A	0	10
MY2A0	39,6722	-7,67185	0	N/A	N/A	N/A	Many
MY35C	39,6621	-7,61378	0	N/A	N/A	N/A	1
MY39A	39,66647	-7,64467	0	N/A	N/A	N/A	1
MY41A	39,66115	-7,65077	0	N/A	N/A	N/A	1
MY43C	39,6652	-7,6652	1	N/A	N/A	N/A	1
MY69A	39,71281	-7,71614	0	N/A	N/A	N/A	1
MY96A	39,67398	-7,65452	0	N/A	N/A	N/A	1
MYA08	39,6792	-7,61518	0	N/A	N/A	N/A	1
MYA43	39,65214	-7,70797	0	N/A	N/A	N/A	1
MZ02A	39,72427	-8,0647	N/A	0	0	N/A	10
NJ62R	40,51678	-8,02062	N/A	0	0	N/A	10
NN25G	40,44484	-7,03726	0	0	0	0	10
NNI30	40,34768	-6,96878	1	1	0	N/A	Many
NU86O	39,73927	-7,40152	N/A	N/A	N/A	0	1
NV13B	40,08389	-7,21972	0	N/A	0	0	10
NV28M	39,78056	-7,25639	N/A	1	N/A	N/A	10
NW0D3	39,832	-8,22092	0	0	0	N/A	10
NW2I4	39,82792	-8,12897	N/A	0	0	N/A	Many
RB05A	39,86303	-8,89726	1	1	1	N/A	Many
RB0D8	39,81924	-8,84867	0	1	1	N/A	Many
RB11A	39,40826	-8,91692	1	1	1	0	Many
RB25C	39,86903	-8,76862	0	0	1	1	Many
RB5J6	39,83595	-8,90603	1	1	1	1	Many

RBF26	39,73319	-8,81036	1	1	1	1	Many
RBS53	39,80707	-8,7007	N/A	1	1	N/A	10
RC06A	39,67212	-8,6	1	1	1	1	Many
RG06G	39,52019	-8,90109	0	N/A	N/A	N/A	10
RG31I	39,5	-9,1036	N/A	0	0	N/A	10
RH60E	39,50125	-8,88004	N/A	N/A	0	N/A	1
RK13B	39,57352	-8,41854	0	0	0	0	Many
RKD87	39,58069	-8,46463	0	N/A	N/A	0	10
RL1J7	39,50883	-8,08578	N/A	0	N/A	N/A	1
RMC12	39,5509	-8,15106	0	0	0	0	Many
RR35A	39,4822	-8,27862	N/A	0	N/A	N/A	1
RYS23	39,2585	-8,88608	1	1	1	0	Many
RZV47	39,22073	-8,69976	N/A	N/A	N/A	0	10
SB69C	39,44592	-8,3991	1	1	0	N/A	Many
SB76C	39,25628	-8,31925	0	0	1	0	Many
SF01A	39,21553	-8,62883	N/A	0	N/A	N/A	1
SG10A	39,0919	-9,3094	N/A	N/A	0	N/A	1
SG34K	39,15522	-9,28135	1	0	0	0	Many
SH5C1	39,08011	-8,99566	N/A	N/A	0	N/A	1
SR14A	38,90749	-8,89998	N/A	N/A	N/A	0	1
SRA27	38,97648	-8,84402	N/A	0	0	N/A	10
SRA75	38,9479	-8,70441	N/A	N/A	N/A	0	1
SS2A1	38,99454	-8,46345	0	0	0	0	Many
SS7B6	38,83019	-8,58831	0	0	N/A	N/A	10
SS91B	39,11047	-8,40228	0	1	0	0	Many
SS96A	38,83019	-8,58831	0	N/A	N/A	N/A	10
SSD82	38,83019	-8,58831	0	N/A	N/A	N/A	10

SSE34	38,96583	-8,69806	0	N/A	0	0	Many
SU19A	38,75652	-9,21238	N/A	N/A	0	N/A	10
SX03A	38,735	-9,29703	N/A	0	N/A	N/A	1
SZ62A	38,71405	-8,91617	0	0	0	0	10
TF3D1	38,73128	-8,77903	1	1	0	0	Many
TF43A	38,65167	-8,635	0	0	0	0	Many
TF74C	38,57882	-8,93967	0	0	0	N/A	Many
VA61C	39,44	-7,46833	1	1	0	0	10
VC12E	39,26871	-7,58112	N/A	N/A	0	N/A	10
VD11L	39,29	-7,47833	0	0	0	N/A	10
VD74B	39,39694	-7,65472	0	0	0	0	Many
VD88G	39,29207	-7,48823	1	1	1	0	Many
VDA02	39,29897	-7,43146	0	N/A	N/A	N/A	1
VE81I	39,2829	-7,96528	0	N/A	N/A	N/A	10
VF91A	39,19602	-7,64548	0	N/A	0	N/A	10
VG49G	39,04333	-7,89333	1	1	0	0	Many
VN09A	38,78943	-8,04707	0	N/A	0	0	10
VN54F	38,72556	-7,98806	N/A	N/A	N/A	0	1
VN82D	38,74169	-8,07738	0	0	0	N/A	Many
VU71H	38,77567	-7,18752	N/A	0	0	0	Many
VW380	38,63095	-8,22532	0	0	N/A	N/A	Many
VW63C	38,64153	-8,19372	0	0	N/A	N/A	10
VX14G	38,66215	-7,98161	N/A	0	0	N/A	10
VX19H	38,56403	-7,90856	0	0	N/A	N/A	10
VX35H	38,5	-7,85	N/A	0	0	N/A	Many
VX94B	38,65	-7,98139	0	0	0	N/A	10
WA62B	38,3719	-8,5086	0	0	0	0	Many

WD16A	38,44972	-7,48167	N/A	N/A	0	0	10
WD39A	38,40628	-7,5811	0	0	0	0	Many
WD48E	38,40762	-7,55352	N/A	0	1	0	Many
WD81B	38,40433	-7,55413	0	0	0	0	Many
WE12A	38,34052	-7,29839	0	0	0	N/A	Many
WF04A	38,13611	-8,49861	N/A	0	N/A	N/A	10
WL59B	38,09938	-7,4079	N/A	0	0	N/A	Many
WL72I	38,08333	-7,22039	0	0	N/A	N/A	10
WP21Y	38,3472	-8,42675	N/A	0	N/A	N/A	10
WS53A	38,035	-7,89836	0	0	0	0	Many
WS81A	37,99167	-7,925	0	0	0	0	Many
WS85B	38,0172	-7,805	N/A	0	0	N/A	10
WS90L	37,85417	-8,09361	N/A	0	N/A	N/A	1
WT32C	37,6238	-7,5252	N/A	0	N/A	N/A	1
WTA04	37,74667	-7,55	N/A	N/A	0	N/A	1
WU36B	37,6555	-8,75583	N/A	N/A	0	N/A	1
WU50Z	37,50722	-8,71667	N/A	N/A	0	N/A	10
WU53A	37,70917	-8,61389	1	1	1	1	Many
WU63A	37,63018	-8,63135	N/A	0	N/A	N/A	1
WU81A	37,63018	-8,63135	0	N/A	N/A	0	10
WV84C	37,75286	-8,33631	N/A	0	0	N/A	10
YD1G0	37,26103	-8,122	1	0	0	0	Many
YE19N	37,49814	-7,51608	1	0	1	0	Many
YF13A	37,23065	-7,47812	N/A	0	0	0	10
YN12K	37,1613	-7,90128	0	0	0	N/A	10

Seasons columns: 0 – absence of the species; 1 – presence of the species; N/A – data not available; Code column: 1 – one performed capture; 10 – ten performed captures; Many – more than ten performed captures.
