# Supplementary material for: Geographical distribution of Culicoides (DIPTERA: CERATOPOGONIDAE) in mainland Portugal: Presence/absence modelling of vector and potential vector species
Source: PLoS One. 2017 Jul 6;12(7):e0180606. doi: 10.1371/journal.pone.0180606 (PMC5500329; doi:10.1371/journal.pone.0180606)
Supplement: S6 Table — (PDF) [file pone.0180606.s007.pdf]

S6 Table. Meteorological data obtained from the closest meteorological stations to the farms.

***Culicoides* species**	**Temperatures (°C)[1]**			**Relative Humidity (%)[2]**		
	**Min**	**Mean[3]**	**Max**	**Min**	**Mean[3]**	**Max**
C. begueti	10.2	23.29	40.4	18	46.73	88
C. circumscriptus	2.6	19.50	40.4	9	58.34	100
C. derisor	12.1	24.00	40.4	11	43.43	100
C. festivipennis	1.5	19.93	40.4	9	52.62	100
C. gejgelensis	-1.1	16.47	36.1	11	63.04	100
C. heteroclitus	6	20.85	40.4	9	50.07	100
C. imicola	-1.5	18.37	40.4	9	58.58	100
C. jumineri near *C. bahrainensis*	4.1	20.81	40.4	9	51.96	100
C. kurensis	5.5	22.16	40.4	9	47.78	100
C. longipennis	6	22.83	40.4	9	46.48	100
C. montanus	-2.8	17.46	39.2	10	62.90	100
C. newsteadi	-3.8	17.3	40.4	9	62.06	100
Nubeculosus group[4]	4.1	20.73	40.4	9	50.34	100
C. obsoletus	-1.7	17.33	39.2	10	62.99	100
C. pulicaris	-3.1	17.67	39.2	9	62.68	100
C. punctatus	-2.6	17.49	40.4	9	59.42	100
C. sahariensis	8.3	23.06	40.4	9	48.67	100
C. scoticus	0.1	16.52	38.6	11	66.01	100
C. semimaculatus	13.8	24.77	40.4	11	41	79
C. subfagineus	6.4	20.85	40.4	9	53.31	100
C. subfasciipennis	-3.8	21.2	38.8	12	50.75	100
C. univittatus	-1.4	14.84	40.4	10	66.72	100

[1]Minimum values were registered in the night of capture and maximum values in the day of trap placement; [2]Relative humidity was registered at 3 pm on the day of trap placement and at 9 am on the day of trap removal; [3]Mean values were obtained from all minimum and maximum values registered in each species captures; [4]Includes *C. nubeculosus*, *C. puncticollis* and *C. riethi* species.
