# Supplementary material for: Geographical distribution of Culicoides (DIPTERA: CERATOPOGONIDAE) in mainland Portugal: Presence/absence modelling of vector and potential vector species
Source: PLoS One. 2017 Jul 6;12(7):e0180606. doi: 10.1371/journal.pone.0180606 (PMC5500329; doi:10.1371/journal.pone.0180606)
Supplement: S7 Table — (PDF) [file pone.0180606.s008.pdf]

S7 Table. Absolute and relative frequencies of estimated and analysed *Culicoides* collected during the NESP for BTD (2005-2013) in mainland Portugal.

***Culicoides* species**	**Total collected (estimated)**	**Total collected (% estimated)**	**Total analysed**	**Total analysed (%)**
C. imicola	3 109 345	70.92	180 832	47.52
Obsoletus group	216 349	4.93	71 216	18.72
C. pulicaris	3 575	0.08	1 811	0.48
C. punctatus	409 361	9.34	62 602	16.45
C. newsteadi	84 759	1.93	26 756	7.03
Other *Culicoides* species	561 114	12.8	37 240	9.83
**Total**	4 384 502	100	380 456	100
